# Autobiographical age awareness disturbance syndrome in autoimmune limbic encephalitis: two case reports

**DOI:** 10.1186/s12883-015-0498-7

**Published:** 2015-11-20

**Authors:** Takeshi Kuroda, Akinori Futamura, Azusa Sugimoto, Akira Midorikawa, Motoyasu Honma, Mitsuru Kawamura

**Affiliations:** Department of Neurology, Showa University School of Medicine, 1-5-8 Hatanodai, Shinagawa-ku, 142-8666 Tokyo Japan; Department of Psychology, Chuo University, 742-1, Higashi-nakano, Hachioji, 192-0393 Tokyo Japan

**Keywords:** Age awareness, Autobiographical memory, Autoimmune limbic encephalitis, NMDA, Medial temporal lobe, Orbitofrontal lobe, Mental time travel

## Abstract

**Background:**

Autobiographical memory is a form of episodic memory characterized by a sense of time and consciousness that enables an individual to subjectively re-experience his or her past. As part of this mental re-enactment, the past is recognized relative to the present. Dysfunction of this memory system may lead to confusion regarding the present perception of time.

**Case presentation:**

Two Japanese women (42 and 55 years old) temporarily believed they were living in their past during a course of autoimmune limbic encephalitis. Their autobiographical memories and behaviour reflected their self-estimated age, and they could not recall memories experienced beyond that age. More surprisingly, their subjective age estimations and autobiographical memories were transiently corrected when they were made aware of their true age. Disorientation, anterograde amnesia, and retrograde amnesia were common additional symptoms. Neuroimaging suggested disturbances in medial temporal and orbitofrontal brain regions in both cases.

**Conclusions:**

This syndrome is characterized by three elements: 1) failure to subjectively recognize the present; 2) inability to suppress irrelevant past memories; and 3) transient restitution of awareness of the present through realization of the individual’s true age. We defined this syndrome as ‘autobiographical age awareness disturbance’, and focused our investigation on the role of age self-awareness. If recall of relevant and suppression of irrelevant past memories are both necessary to subjectively recognize the present relative to the past, dysfunction of medial temporal and orbitofrontal brain regions is predicted to lead to abnormal subjective placement in time. However, the subjective experience of age tends to be an important informational component for retrieving remote autobiographical memories. This suggests that correct age awareness is essential for the proper recognition of the remote past in relation to the present. This is the first report to focus on the relationship between subjective temporal orientation and age self-awareness. While the role of age awareness in this process is still unclear, investigating autobiographical age awareness disturbance as a part of subjective temporal awareness dysfunction can be useful in understanding the processes underlying human time recognition.

## Background

Autobiographical memory (ABM) is a form of episodic memory affecting subjective orientation with respect to instances of ‘what’, ‘where’, and ‘when’. It is defined as a system that ‘receives and stores information about temporally dated episodes or events’ [1]. ABM is also characterized by a subjective sense of time and noetic experiences. The system allows for figurative ‘mental time travel’ from the present to the past, allowing one to re-live (through autonoetic awareness) personal previous experiences [2]. As part of this figurative time travel, the present is recognized relative to the past. Dysfunction of this memory system may lead to confusion of temporal orientation.

Results of recent studies suggest that the medial temporal lobe plays crucial roles in long-term encoding and retrieval of detailed ABM content [3, 4]. Limbic encephalitis is one of the diseases that affects medial temporal lobe and is characterised by an acute or sub-acute onset of memory disorder, associated with seizures and psychiatric manifestations. Limbic encephalitis can be caused by infections or result from autoimmune aetiology. Many patients with autoimmune limbic encephalitis have inflammatory markers in the cerebrospinal fluid with (or without) anti-neuronal antibodies [5]. These antibodies are directed against two broad categories of antigens: intracellular or classic paraneoplastic antigens, including Hu, Ma2, CV2/CRMP5, and amphiphysin, and cell membrane antigens, including voltage-gated potassium channels, N-methyl-d-aspartate (NMDA) receptor, and other antigens expressed in the neuropil of the hippocampus and cerebellum [5].

The two cases of autoimmune limbic encephalitis are discussed here, anti-NMDA receptor titres were elevated in both serum and cerebral spinal fluid. In the course of the disease, patients temporarily believed they were living in their past. Their ABM and behaviour reflected their self-estimated ages, and they could not recall memories experienced after those ages. Such symptoms in limbic encephalitis were previously explained as resulting from a collapse of reality filtering and described as a special type of confabulation [6]. However, surprisingly in our cases, their ABM was transiently corrected when they were made aware of their true age. We previously reported a case of autobiographical amnesia and suggested that age awareness may play a crucial role in ABM retrieval [7]. To understand this unique phenomenon, we additionally investigated the role of perceived age in ABM retrieval using a simple questionnaire. The results suggested that perceived age may constitute important temporal context information when retrieving remote ABMs. We discussed the role of age self-awareness in human time recognition through characterization of this unique syndrome, which we define as ‘autobiographical age awareness disturbance (AAAD)’.

## Case presentation

### Case 1

#### Initial symptoms

A 42-year-old Japanese woman, with a junior college education level, initially had a headache and slight fever (day 0). On day 2, after breakfast, she had a generalized, tonic-clonic seizure, beginning with her left jaw. On day 9, her linguistic ability spontaneously changed to that of an approximate 5-year-old, and she behaved in a childish manner, demanding that her mother serve her ‘gyoza’ (a favourite dish among Japanese children). She sometimes made pronunciation errors typical of a child, such as using the word ‘dechu’ to represent the Japanese copula ‘desu’. She was hospitalized on day 12. On admission, she showed signs of spatial and temporal disorientation. Retrograde and anterograde amnesia were also present. She did not remember a large earthquake that occurred 2 years ago in Tohoku, Japan. She also forgot the doctor she met and the examination that he performed in the morning. A standard bedside neurological evaluation revealed normal functioning of her cranial nerves, motor and sensory functions, and coordination. Bradycardia was present during sleep, possibly due to autonomic nerve dysfunction. She had no special medical and family history or relevant past interventions.

#### Characterization of AAAD syndrome

During hospitalization, the patient told us on one instance that she was 17 years old, and currently in her high school classroom. When she perceived herself as a 17-year-old, she spoke and laughed in the manner of a Japanese teenager. During this time, she was able to recognize her parents, but she had no apparent memories of her husband or children. She could not remember where and when she met her husband, or when she got married. On the other hand, she remembered the elementary school and junior high school from which she graduated. However, the woman was informed of her correct age, she reacted with surprise, and replied that it was strange that she was still in high school. Eventually the woman recalled that she was 42 years old, and was able to remember her marriage and three children.

**Fig. 1:**
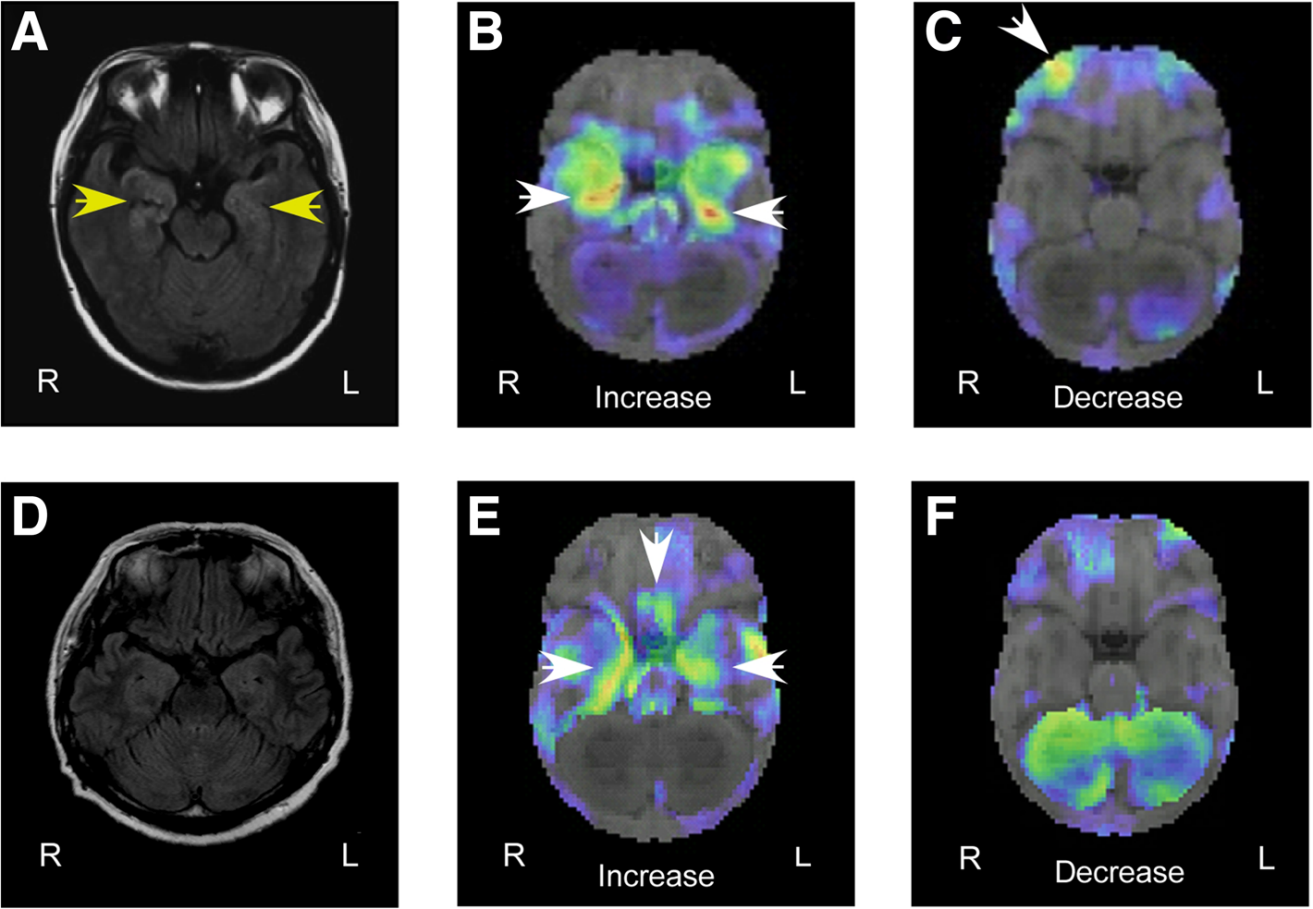
Imaging data. Case 1 (**a–c**). High-intensity signal in bilateral medial temporal lobes as shown by magnetic resonance imaging, axial fluid-attenuated inversion recovery image (**a**). Increased blood flow in bilateral medial temporal lobes (**b**), with decreases in the right orbitofrontal cortex (**c**), as assessed by ^123^I-IMP brain perfusion single-photon emission computed tomography with three-dimensional stereotactic surface projection analysis. Case 2 (**d**–**f**). No specific findings on the magnetic resonance imaging, axial fluid-attenuated inversion recovery image (**d**). Increased blood flow in bilateral medial temporal lobes and the posterior orbitofrontal cortex (**e**), with no decreases (**f**), as shown by ^123^I-IMP brain perfusion single-photon emission computed tomography with three-dimensional stereotactic surface projection analysis

Her mini-mental state examination (MMSE) score was 15 (out of 30). There was also a reduction in verbal and general memory quotient (MQ) scores and delayed recognition, as assessed by the Wechsler memory scale-revised (WMS-R). A decrease in general, verbal and performance intelligence quotient (IQ) scores was also observed, as assessed by the Wechsler adult intelligence scale (WAIS)-III. A cerebral spinal fluid (CSF) analysis revealed a cell density of 20 per mm^3^ (consisting of predominantly lymphocytes) with normal protein levels. There was no elevation in antibody titres of herpes simplex virus (HSV), varicella zoster virus (VZV) and Epstein-Barr virus (EBV). Similar results were found for HSV deoxyribonucleic acid using polymerase chain reaction (PCR). A blood examination revealed normal findings, aside from slightly elevated C-reactive protein. Anti-NMDA receptor titres were elevated in both serum and CSF. Fluid-attenuated inversion recovery (FLAIR) brain magnetic resonance imaging (MRI) showed bilateral, high-intensity signals in the medial temporal lobe (Fig. 1a). Single-photon emission computed tomography (SPECT) using ^123^I-IMP brain perfusion showed that blood flow was bilaterally increased in the medial temporal lobe and decreased in the right orbitofrontal cortex (Fig. 1b and c). No ovarian teratoma was detected via enhanced pelvic MRI. A sporadic, right-temporal-lobe-dominant spike discharge and diffuse slow waves were visible by electroencephalography (EEG). After evaluation, the patient was diagnosed with anti-NMDA receptor encephalitis and was treated with intravenous high-dose corticosteroids (methylprednisolone, 1 g/day for 3 days), immunoglobulin therapy (0.4 g/kg/day for 5 days), and oral anti-epileptic drugs (levetiracetam, 3 g/day). The treatments improved all AAAD-related symptoms, such as disorientation and amnesia, and the patient was discharged from the hospital on day 81. The performance in WMS-R and WAIS-III administered 2 months after discharge significantly improved up to the normal range. There was no abnormal hyperperfusion in the medial temporal lobe or hypoperfusion in the right orbitofrontal cortex in ^123^I-IMP brain perfusion SPECT performed 9 months later.

### Case 2

#### Initial symptoms

A 55-year-old Japanese woman who was employed as a part-time childcare assistant initially experienced a slight sore throat (day 0). She reported being under stress caused by the daily problems of looking after her elderly parents. On day 8, she became emotionally unstable and required supervision by her daughter all day. She lost all awareness of time and frequently checked the television display and clock. She mistook night for day and was unable to tell the date. In the evening, she was unable to carry out simple cooking tasks, and asked her daughter for help. On day 9, she was conscious but unable to walk as a result of involuntary limb movement; she also repeatedly recalled old memories and expressed concern about her parents. On day 11, she was admitted to a psychiatric hospital, but was transferred to our facility due to suspected encephalitis. At the time of admission, she showed signs of instability in cognition and attention to her surroundings. Although she retained verbal ability, she was disoriented with respect to time and place. Retrograde and anterograde amnesia were also present; she could not recall the work she was engaged in or when and how she was admitted to our hospital. Spontaneous tonic spasms appeared in her limbs bilaterally. A standard bedside neurological examination revealed otherwise normal findings for her cranial nerves, motor and sensory functions, and coordination. She had no special medical and family history or relevant past interventions.

#### Characterization of AAAD syndrome

During her hospitalization, the patient informed us that she believed she was 27 years old. She informed us that she was currently living in Aomori Prefecture and working as a receptionist, which accurately reflected events she experienced at age 27. She could recall memories of her marriage at age 25, but not her daughter, whom she gave birth to at 27. On a separate occasion, the patient adopted a birthing posture, as if she was re-experiencing the delivery that she had gone through when she was 27. However after informing her of her true age (55 years old), she recognized this fact, and her memory was transiently corrected accordingly.

Her MMSE score was 26. She showed decreased measures of attention and concentration, along with delayed recognition, as assessed by the WMS-R; however, her WAIS-III evaluation was normal. A CSF analysis revealed normal cell density, protein, and glucose levels. Antibody titres to HSV, VZV, and EBV were also normal, and HSV was undetected by PCR. Subsequently, elevated serum anti-NMDA receptor antibody titres were detected in both serum and CSF.

There were no abnormal findings revealed by brain MRI (Fig. 1d). However, ^123^I-IMP brain perfusion SPECT showed increased blood flow bilaterally in the medial temporal lobes and orbitofrontal cortex (Fig. 1e and f). Epileptic discharges and abnormal slow waves were not detected by EEG. Following evaluation, she received a diagnosis of anti-NMDA receptor encephalitis. Oral anti-epileptic drugs (carbamazepine, 200 mg/day; levetiracetam, 2 g/day) and intravenous high-dose corticosteroid therapy (methylprednisolone, 1 g/day for 3 days) improved her symptoms, confirming the diagnosis of AAAD. The patient was discharged on day 51. At the time of discharge, there was no disorientation or amnesia, her MMSE score improved to 28/30. Subsequently, she stopped coming to our hospital and we were unable to perform follow-ups.

Clinical findings for both cases are summarized in Table 1.

**Table 1:** Clinical findings of cases

Case	1	2
Age	42	55
Sex	Female	Female
Diagnosis	Anti-NMDA receptor encephalitis	Anti-NMDA receptor encephalitis
Neuropsychological symptoms		
AAAD (wrong age estimation)	Yes (17)	Yes (27)
Disorientation	Yes	Yes
Retrograde and anterograde amnesia	Yes	Yes
Neuropsychological examination		
MMSE^a^	15/30	26/30
WMS-R^b^		
Verbal MQ	<50	94
Visual MQ	111	97
General MQ	71	94
Attention/Concentration	97	79
Delayed recognition	64	75
WAIS-III^b^		
Verbal IQ	82	94
Performance IQ	68	86
Full scale IQ	72	89
Clinical examination		
CSF	20 cells/mm^3^ (lymphocytes predominant) with normal protein levels	Normal cells, protein levels
	Elevated anti-NMDA receptor antibody titres	Elevated anti-NMDA receptor antibody titres
Blood	Elevated anti-NMDA receptor antibody titres	Elevated anti-NMDA receptor antibody titres
Brain MRI	High intensity bilateral medial temporal lobe lesion (assessed by FLAIR)	No specific findings
^123^I-IMP brain perfusion SPECT	Increase in bilateral medial temporal lobe blood flow; decrease in right orbitofrontal region blood flow	Bilateral increase in medial temporal lobe and orbitofrontal region blood flow
EEG	Sporadic spike discharge and diffuse slow waves	No epileptic discharge

^a^Score ≥ 28 points (out of 30) indicates normal cognition in their age [14]

^b^The index scores of the Wechsler memory scale-revised and Wechsler adult intelligence scale-III have a mean of 100, and standard deviation of 15

### Discussion of cases

We defined the syndrome experienced in the two cases as AAAD, a specific group of symptoms in which patients temporarily believe they are chronologically younger, accompanied by general ABM disturbances. In both cases, the patients misidentified their own ages and behaved accordingly as younger individuals. Although their ABM content was accurate, they could not recall memories beyond their respective estimated ages. Interestingly, patient mistakes in age estimation could be corrected, which updated their ABM content to the present. We defined AAAD as having three elements: 1) failure to subjectively recognize the present; 2) inability to suppress irrelevant past memories; and 3) transient restitution of awareness of the present through realization of the individual’s true age.

Our observations suggest that the primary cause of failure to subjectively recognize the present was a disturbance in ABM retrieval. ABM allows for figurative ‘mental time travel’ from the present to the past, allowing one to re-live personal previous experiences [2]. As part of this process, the present is recognized relative to the past. Recent studies suggest that the medial temporal lobe and hippocampus play crucial roles in long-term encoding and retrieval of detailed ABM content [3, 4]. In the present report, an abnormal high-intensity lesion was visualized by brain MRI in one patient, while abnormal blood medial temporal lobe flow was evident by brain perfusion SPECT in both cases. These imaging data indicate medial temporal lobe dysfunction, and accordingly, anterograde and retrograde amnesia were additional symptoms in our patients. Two important theoretical approaches describing the role of the hippocampus in ABM retrieval are the standard model of consolidation and the multiple trace theory [8, 9]. The standard model suggests that hippocampal function in ABM is time-limited and memories become gradually independent of the medial temporal lobe over a period of time. In contrast, the multiple trace theory predicts that the process of recalling autobiographical memories requires the hippocampal formation irrespective of how old the relevant memories are. Remote autobiographical memory deficits due to hippocampal damage, such as the ones observed in the cases described here, support the multiple trace theory, a life-long role of the hippocampus in memory storage and retrieval.

Orbitofrontal region brain dysfunction is a hypothesized cause of inability to suppress irrelevant past memories. The anterior limbic system (especially the posterior orbital frontal cortices) aids in filtering perceptions to allow individuals to determine whether an activated memory is part of the present compared with the past [10]. This system signifies when an activated memory does not pertain to on-going reality, and prevents behaviour from being based on imagined events [11]. Dysfunction of this reality filtering system sometimes results in spontaneous behavioural confabulation: individuals act according to false ideas generally traced back to real experiences and justify their actions with invented stories [6]. In both of our patients, abnormal blood flow in the orbitofrontal region was visible by brain perfusion SPECT, suggesting dysfunction. Previous reports reveal that the pattern of brain perfusion in anti-NMDA receptor encephalitis is variable; either increased or decreased perfusion is detected in the frontal and temporal lobes [12, 13]. It has also been previously reported that disruption of reality filtering could occur owing to limbic encephalitis [6].

**Fig. 2:**
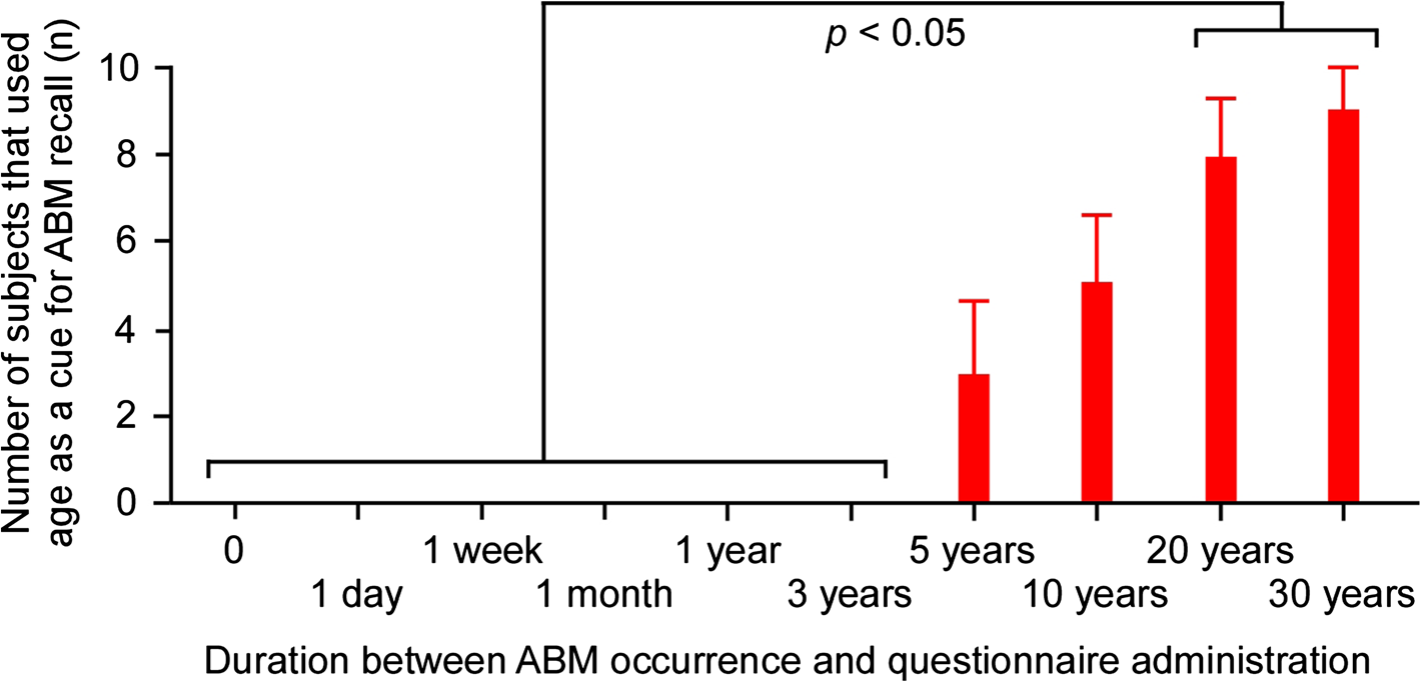
Autobiographical memory questionnaire results. Age tended to be the technique used by most participants to recall autobiographical memories older than 5 years. A one-way analysis of variance showed a significant main effect of past period (*F*_(9,81)_ = 19.23, *p* < 0.0001). A post-hoc test revealed that the incidence of subjects using age to recall events was significantly increased for events that occurred 20 and 30 years prior to testing, compared with events that had occurred on the day of testing through to 3 years prior (*p* < 0.05 for all values)

More interestingly, in the two cases presented here, restoration of each patient’s current age resulted in a transient update of their respective ABM content. To understand this unique phenomenon, we investigated the role of perceived age in ABM retrieval using a simple questionnaire. In this questionnaire, we asked 10 healthy subjects (mean age: 48.4 years; standard deviation [S.D.]: 11.9) to recall any ABM from the following: the day the questionnaire was administered, the day before testing, and 1 week, 1 month, 1 year, 3 years, 5 years, 10 years, 20 years, or 30 years prior, respectively. Subjects were required to verbally recall their ABM, and to describe the whole recall process. For example, when a 65-year-old woman was asked to recall a personal memory from 5 years ago, she answered ‘hmm…5 years ago…when I was 60…let me see, it was the year when I retired…I was grateful that my workmates threw me a resignation party’. As a result, we determined that age association tended to be the technique used by most participants to recall ABMs older than 5 years, and the frequency of this strategy significantly increased for events that occurred 20 and 30 years prior to testing (Fig. 2). This finding suggests that perceived age may constitute important temporal context information when retrieving remote ABMs.

## Conclusions

**Fig. 3:**
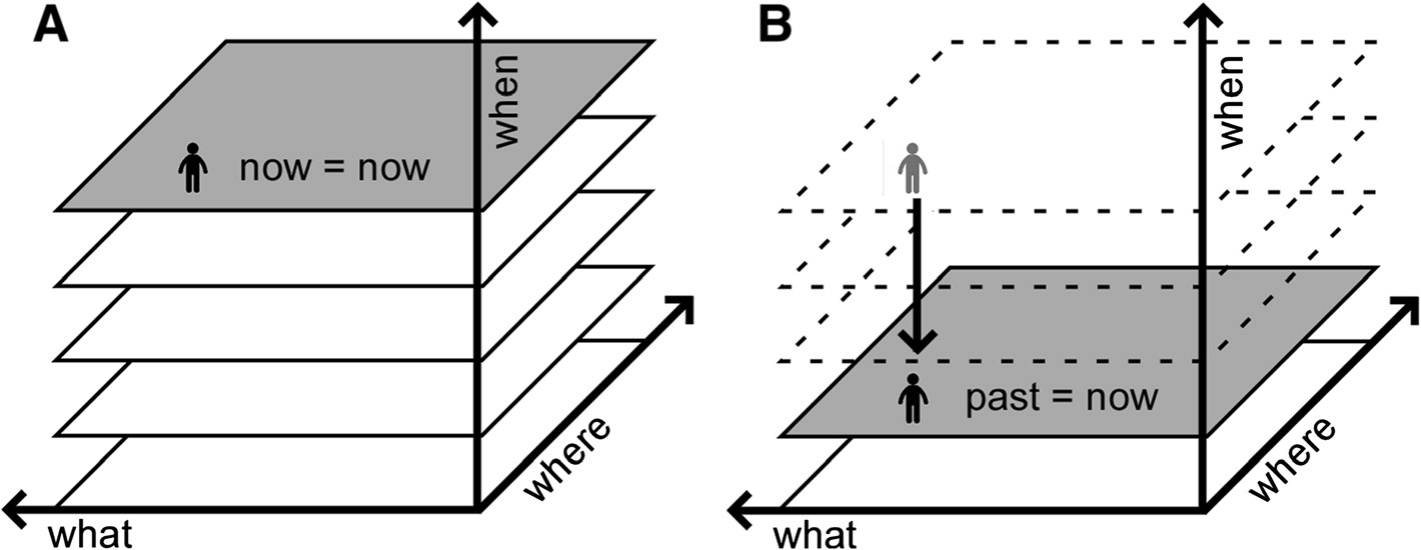
Human time recognition model based on age self-awareness. Normal age awareness: recognizing the subjective present as such (**a**). Autobiographical age awareness disturbance: the subjective ‘past’ becoming ‘the present’ (**b**)

If recall of relevant and suppression of irrelevant past memories are both necessary to recognize the present relative to the past, dysfunctions of medial temporal and orbitofrontal brain regions are expected to cause abnormalities in subjective temporal orientation. However, our observations indicate that perceived age tends to be an important informational cue used to retrieve remote ABMs. Thus, our combined findings suggest that accurate subjective age awareness is essential for proper perception of the present relative to the remote past (see Fig. 3). While the precise role of subjective age awareness in ABM is still unclear, investigating AAAD as a part of this process can contribute to an understanding of the mechanisms underlying human time recognition.

## Consent

Written informed consent was obtained from the patients for publication of this Case Report and any accompanying images. A copy of the written consent is available for review by the Editor-in-Chief of this journal.

## Abbreviations

AAAD: Autobiographical age awareness disturbance
ABM: Autobiographical memory
CSF: Cerebral spinal fluid
EBV: Epstein-Barr virus
EEG: Electroencephalography
FLAIR: Fluid-attenuated inversion recovery
HSV: Herpes simplex virus
IQ: Intelligence quotient
MMSE: Mini-mental state examination
MRI: Magnetic resonance imaging
MQ: Memory quotient
NMDA: *N*-methyl-D-aspartate
PCR: Polymerase chain reaction
SPECT: Single-photon emission computed tomography
VZV: Varicella zoster virus
WAIS: Wechsler adult intelligence scale
WMS-R: Wechsler memory scale-revised
